# Antibiotic Consumption and Healthcare-Associated Infections Caused by Multidrug-Resistant Gram-Negative Bacilli at a Large Medical Center in Taiwan from 2002 to 2009: Implicating the Importance of Antibiotic Stewardship

**DOI:** 10.1371/journal.pone.0065621

**Published:** 2013-05-30

**Authors:** I-Ling Chen, Chen-Hsiang Lee, Li-Hsiang Su, Ya-Feng Tang, Shun-Jen Chang, Jien-Wei Liu

**Affiliations:** 1 Department of Pharmacology, Kaohsiung Chang Gung Memorial Hospital, Kaohsiung, Taiwan; 2 Infection Control, Kaohsiung Chang Gung Memorial Hospital, Kaohsiung, Taiwan; 3 Division of Infectious Diseases, Department of Internal Medicine, Kaohsiung Chang Gung Memorial Hospital, Kaohsiung, Taiwan; 4 Chang Gung University College of Medicine, Kaohsiung, Taiwan; 5 Department of Kinesiology, Health and Leisure Studies, National University of Kaohsiung, Kaohsiung, Taiwan; Iowa State University, United States of America

## Abstract

**Background:**

Better depicting the relationship between antibiotic consumption and evolutionary healthcare-associated infections (HAIs) caused by multidrug-resistant Gram-negative bacilli (MDR-GNB) may help highlight the importance of antibiotic stewardship.

**Methodology/Principal Findings:**

The correlations between antibiotic consumption and MDR-GNB HAIs at a 2,700-bed primary care and tertiary referral center in Taiwan between 2002 and 2009 were assessed. MDR-GNB HAI referred to a HAI caused by MDR-Enterobacteriaceae, MDR-*Pseudomonas aeruginosa* or MDR-*Acinetobacter* spp. Consumptions of individual antibiotics and MDR-GNB HAI series were first evaluated for trend over time. When a trend was significant, the presence or absence of associations between the selected clinically meaningful antibiotic resistance and antibiotic consumption was further explored using cross-correlation analyses. Significant major findings included (i) increased consumptions of extended-spectrum cephalosporins, carbapenems, aminopenicillins/β-lactamase inhibitors, piperacillin/tazobactam, and fluoroquinolones, (ii) decreased consumptions of non-extended-spectrum cephalosporins, natural penicillins, aminopenicillins, ureidopenicillin and aminoglycosides, and (iii) decreasing trend in the incidence of the overall HAIs, stable trends in GNB HAIs and MDR-GNB HAIs throughout the study period, and increasing trend in HAIs caused by carbapenem-resistant (CR) *Acinetobacter* spp. since 2006. HAIs due to CR-*Acinetobacter* spp. was found to positively correlate with the consumptions of carbapenems, extended-spectrum cephalosporins, aminopenicillins/β-lactamase inhibitors, piperacillin/tazobactam and fluoroquinolones, and negatively correlate with the consumptions of non-extended-spectrum cephalosporins, penicillins and aminoglycosides. No significant association was found between the increased use of piperacilllin/tazobactam and increasing HAIs due to CR-*Acinetobacter* spp.

**Conclusions:**

The trend in overall HAIs decreased and trends in GNB HAIs and MDR-GNB HAIs remained stable over time suggesting that the infection control practice was effective during the study period, and the escalating HAIs due to CR- *Acinetobacter* spp. were driven by consumptions of broad-spectrum antibiotics other than piperacillin/tazobactam. Our data underscore the importance of antibiotic stewardship in the improvement of the trend of HAIs caused by *Acinetobacter* spp.

## Introduction

Infections caused by multidrug-resistant (MDR) Gram-negative bacilli (GNB) poses a threat to affected patients worldwide [1]. Some clinically important MDR-GNBs including extended-spectrum cephalosporin-resistant Enterobacteriaceae (e.g., *Escherichia coli*, *Enterobacter* species and *Klebsiella pneumoniae*), MDR-*Pseudomonas aeruginosa*, and carbapenem-resistant (CR) *Acinetobacter* spp. are of particular concern [2], as more than 50% of these GNB species that caused healthcare-associated infections (HAIs) have been reported to be MDR [3]. Compared with infections due to the antibiotic-susceptible GNB counterparts, MDR-GNB infections frequently lead to poorer outcomes such as longer hospital stays, increased mortality, and higher hospitalization cost [4]. It has been well documented that the selective pressure resulting from non-prudent antibiotic consumption is the major cause of the increasing emergence of MDR pathogens [1], [2].

A substantial number of reports demonstrated the relationships between antibiotic consumptions and the emergences of MDR-GNB in hospital settings [5]-[10]. However, to our knowledge, so far there has not been a single study that specifically designed to explore the dynamics of antibiotic consumptions and the incidence of MDR-GNB HAI. The objectives of this study were (i) to understand the trends in antibiotic consumption and incidence of HAIs, and (ii) to clarify the relationships between antibiotic consumptions and the evolutionary MDR-GNB HAIs during an eight-year period at a large medical center in Taiwan. The implications of this study will be discussed.

## Methods

This study analyzed antibiotic consumptions in adult patients and the incidences of antimicrobial resistance among clinically significant pathogens for HAIs between January 2002 and December 2009 at Kaohsiung Chang Gung Memorial Hospital (KSCGMH), a 2,700-bed facility that serves as a primary care and tertiary referral center in Taiwan. The study was conducted with a waiver of informed consent from the participants, which was approved by the Institutional Review Board (Ethics Committee) of Chang Gung Memorial Hospital (Document no. 97-1694B).

Consumed oral and parenteral antibiotics that were retrieved from the electronic database of the hospital’s pharmacy for analyses included: carbapenems (imipenem, meropenem, and ertapenem), non-extended-spectrum cephalosporins (cefazolin, cefuroxime), extended-spectrum cephalosporins (ceftriaxone, ceftazidime, cefpirome, and cefepime), natural penicillin (penicillin G), aminopenicillins (ampicillin and amoxicillin), ureidopenicillin (piperacillin), aminopenicillins/β-lactamase inhibitor (amoxicillin/clavulanate and ampicillin/sulbactam), anti-pseudomonal penicillin/β-lactamase inhibitor (piperacillin/tazobactam), aminoglycosides (gentamicin and amikacin), fluoroquinolones (ciprofloxacin, levofloxacin, and moxifloxacin), folate pathway inhibitors (trimethoprim-sulfamethoxazole), and glycopeptides (vancomycin and teicoplanin). Antibiotic consumption was evaluated based on the defined daily dose (DDD) per 1,000 inpatient days for each prescribed antibiotic [11] and the quarterly categorized prescription to which the antibiotic belonged. The hospital inpatient days were obtained from the institute’s administrative database. The annual hospital inpatient days at KSCGMH increased from 641,212 in 2002 to 703,111 in 2009.

HAIs were defined as infections that were not present and without evidence of incubation at the time of admission to KSCGMH, and were identified based on the CDC diagnostic criteria for nosocomial infections [12] at regular surveillance for HAIs between January 2002 and December 2009. Specific HAIs and the pathogen(s) were identified according to the CDC diagnostic criteria as well, and some of the HAIs were polymicrobial infections [12]. The regular surveillance of HAI during the study period at KSCGMH was performed by the same staff that consisted of senior infection-control practitioners under the supervision of a senior infectious-diseases specialist (Dr. JW Liu).

A HAI-GNB was defined as a GNB that was judged to be the pathogen of a HAI. HAI-GNBs tracked in this study included E. coli, K. pneumoniae, K. oxytoca, Enterobacter cloacae, Serratia marcescens, Proteus spp., P. aeruginosa, Acinetobacter spp., Stenotrophomonas maltophilia, and other glucose-nonfermenting GNBs (i.e., P. fluorescens, P. putida, Burkholderia cepacia, Chryseobacterium meningosepticum, C. indologenes, and Alcaligenes spp.). MDR was defined based on the criteria proposed by Magiorakos et al [13] with modifications. As a large number of GNBs are intrinsically multidrug-resistant, a MDR-HAI-GNB was defined in this study as a MDR-Enterobacteriaceae (other than Salmonella spp. and Shigella spp.), a MDR-P. aeruginosa, or a MDR-Acinetobacter sp. that was identified as a pathogen for HAI. A MDR-Enterobacteriaceae referred to an Enterobacteriaceae isolate that was resistant to ≥1 agent in 3 or more of the following antimicrobial classes: aminoglycosides, carbapenems, non-extended-spectrum cephalosporins, extended-spectrum cephalosporins, antipseudomonal penicillin/β-lactamase inhibitor, fluoroquinolones, folate pathway inhibitors, tigecycline, aminopenicillins /β-lactamase inhibitors, polymyxins, and tetracyclines [13]. A tested antibiotic profile for glucose-nonfermenting GNB adopted at KSCGMH included aminoglycosides, anti-pseudomonal carbapenems (imipenem and meropenem), anti-pseudomonal cephalosporins, anti-pseudomonal fluoroquinolines, piperacilliln/tazobactam, monobactam, and polymixycins. A MDR-P. aeruginosa referred to a P. aeruginosa isolate that was resistant to ≥1 agent in 3 or more of the antibiotic classes included in the tested antibiotic profile for glucose-nonfermenting GNB [13]. A MDR-Acinetobacter sp. referred to an Acinetobacter isolate that was resistant to ≥ 1 agent in 3 or more of the antibiotic classes including those listed in the tested antibiotic profile for glucose-nonfermenting GNB, and extended-spectrum cephalosporins, folate pathway inhibitors, ampicillin/sulbactam, polymyxins and tigecycline [13].

As a rule, blood and/or specimen of the clinically suspicious infection site, regardless of whether the infection was acquired from hospital settings, was sampled for culture before starting antimicrobial therapy. Blood cultures were incubated in a Bactec 9240 instrument (Becton Dickinson Diagnostic Systems, Sparks, MD) at 35°C for 5 days. Specimens harvested from infection sites other than blood were inoculated in proper culture media which were then incubated at 35°C in ambient air. All plated media were examined for macroscopic evidence of bacterial growth 24h later. If no visible growth was found, the culture plates were further incubated, and the plates were examined daily for 4 consecutive days in case of culture of specimens sampled from normally sterile sites such as blood and cerebrospinal fluid [14]. All bacterial isolates were identified using biochemical tests and were then verified by a Vitek System (bioMérieux, France) using a GNI Card. The susceptibility testing was performed on a daily-service basis using Kirby-Bauer disk-diffusion methods [15]. The tested GNB were classified as susceptible or resistant (including intermediate) strains as recommended by CLSI (previous NCCLS) [16]. Interpretations of MICs of tigecycline against *Acinetobacter* spp. were based on the US-FDA susceptibility breakpoints for Enterobacteriaceae [17]. Data concerning HAIs were expressed as incidence density per 1,000 patient-days/quarter.

Consumptions of each individual antibiotic and incidence of HAIs were first evaluated for trend over time using Box-Jenkins autoregressive-integrated moving average (ARIMA) modeling method [18], [19]. ARIMA models were fit for every series using the standard approach of identification, estimation, and checking. All the series are stationary as were confirmed by augmented Dickey-Fuller tests. The autocorrelation, partial autocorrelation, and inverse autocorrelation functions were used to exclude serial dependencies and to ensure appropriateness of the model. A transfer function was estimated, and an ARIMA model was fit to the remaining noise. Akaike’s information criterion was used to identify the appropriate model by minimizing both the residual variance and the number of parameters [19]. A trend was regarded as statistically significant when a *P* was <0.05; otherwise, it was considered stable.

The presence or absence of associations between selected clinically meaningful antibiotic resistance and consumption series was further explored in pairs using cross-correlation analysis [20], where quarterly time lags of up to one year in both directions (i. e., time lag of −4 to 4) were applied to the antibiotic resistance series [20], and the strength of association was measured by a nonparametric Spearman coefficient. An association was considered significantly present at any quarterly time lag when a *P* was <0.05 and a correlation coefficient *R* was ≥0.6 or ≤−0.6. The highest correlation coefficient for each pair of specific antibiotic consumption and selected clinically meaningful antibiotic resistance determined the most likely positive time lag where the consumption of this antibiotic led to the development of antibiotic resistance or vice versa for that particular pair. A negative time lag for any result indicated that the antibiotic resistance had preceded the consumption of a specific antibiotic and vice versa for a positive time lag. All statistical analyses were performed using SAS software version 9.2 for Window (SAS Institute, Inc., Cary, NC, USA).

## Results

The trends in antibiotic consumptions and the HAI incidence during 2002 and 2009 are summarized Table 1. We found (i) increasing trends in consumptions of extended-spectrum cephalosporins, carbapenems, fluoroquinolones, aminopenicillins/β-lactamase inhibitors, and piperacillin/tazobactam, (ii) decreasing trends in the consumptions of non-extended spectrum cephalosporins, natural penicillins, aminopenicillins, ureidopenicillin, folate pathway inhibitors and aminoglycosides, (iii) stable trend of consumption of glycopeptides, (iv) decreasing trends in overall antibiotic consumptions and incidence of overall HAIs.

**Table 1:** Trends in antibiotic consumption (DDD/1,000 inpatient-days/quarter) and incidences of healthcare-associated infections (events/1,000 inpatient-days/ quarter) from 2002 to 2009 at KSCGMH.

	Mean	Coefficient	*P* value	Transformation	AR or MA^a^ (order b)	*R^2^*	Trend c
**Antibiotic consumption**							
Non-extended-spectrum cephalosporins	184.9	−3.17	<0.01			0.95	Decreasing
Extended-spectrum cephalosporins	67.8	0.96	<0.01			0.83	Increasing
Aminoglycosides	62.2	−0.02	0.01	Log	AR (1)	0.99	Decreasing
Natural penicillin and aminopenicillins	10.0	−0.03	<0.01	Log		0.74	Decreasing
Ureidopenicillins	117.2	<−0.01	0.05	Log	AR (1), MA (2)	0.72	Decreasing
Carbapenems	23.8	0.75	<0.01		AR (1)	0.76	Increasing
Fluoroquinolones	39.9	0.96	<0.01			0.72	Increasing
Glycopeptides	35.2	−0.02	0.81			<0.01	Stable
Folate pathway inhibitors	24.4	−0.02	<0.01	Log		0.83	Decreasing
Aminopenicillins/β-lactamase inhibitors	59.4	1.40	<0.01		AR (1)	0.83	Increasing
Piperacillin/tazobactam	7.5	0.47	<0.01			0.7	Increasing
Overall antibiotics	725.8	−3.83	<0.01			0.65	Decreasing
**Incidence**							
Overall HAIs	4.68	−0.03	<0.01			0.31	Decreasing
GNB HAIs	2.65	−0.02	<0.01			0.25	Stable
MDR-GNB HAIs	0.98	−0.01	0.01			0.2	Stable

Abbreviation: DDD =  defined daily dose; HAI: healthcare associated infection; GNB: Gram-negative bacilli; MDR: multidrug-resistant.

a AR, autoregressive, representing the past values of the quarterly incidence; MA, moving average, representing the past variance of the quarterly observations.

b Delay in quarters before the effect is observed.

c A significant trend referred to that with a *R^2^*>0.3 and *P*<0.05.

A total 12,567 non-duplicated HAI-GNB isolates identified as pathogens in the overall HAIs and specific HAIs are shown in Table 2. Among these HAI-GNB isolates, *E. coli* was most commonly encountered (n* = *3,653 [29.1%]), followed by *P. aeruginosa* (n = 2,340 [18.6%]), and *K. pneumoniae* (n = 1,729 [13.8%]). A total of 4,625 non-duplicated MDR-GNB isolates, accounting for 36.8% of the overall HAI-GNB, were found, which included MDR-Enterobacteriaceae (n = 3,177 [68.7%]), MDR-*Acinetobacter* spp. (n = 1,160 [25.1%]), and MDR-*P. aeruginosa* (n = 288 [6.2%]).

**Table 2:** Numbers of healthcare-associated infection (HAI) and the pathogenic Gram-negative bacilli from 2002 to 2009 at KSCGMH.

HAI	Pathogens					
	*Acinetobacter* spp.	*E. coli*	*K. pneumoniae*	*P. aeruginosa*	Others†	Subtotal, n (%)*
BSI	228	499	417	277	715	2136 (17.0%)
CLABSI	212	75	83	177	196	743 (5.9%)
GI	22	47	38	25	95	227 (1.8%)
Pneumonia	316	57	148	343	287	1151 (2.3%)
SSI	122	319	194	346	702	1683 (13.4%)
SST	27	32	32	51	73	215 (1.7%)
UTI	483	2613	801	1070	1324	6291 (50.1%)
Miscellaneous!	22	11	16	51	21	121 (1.0%)
Subtotal, n (%)*	1432 (11.3%)	3653 (29.1%)	1729 (13.8%)	2340 (18.6%)	3413 (27.2%)	Total, N* = *12567

**Abbreviations:** BSI: bloodstream infection; CLABSI: central-line associated bloodstream infection; GI: gastrointestinal system infection; SSI: surgical site infection; SST: skin and soft tissue infection; UTI: urinary tract infection.

* Derived from n/N X 100.

† Including K. oxytoca, Enterobacter cloacae, Serratia marcescens, Proteus spp., Stenotrophomonas maltophilia, and other glucose-nonfermenting GNB (such as P. fluorescens, P. putida, Burkholderia cepacia, Chryseobacterium meningosepticum, C. indologenes, and Alcaligenes spp.).

! Including bone and joint infection, cardiovascular system infection, central nervous system infection, eye, ear, nose, throat or mouth infection, and reproductive tract infection.

**Figure 1:**
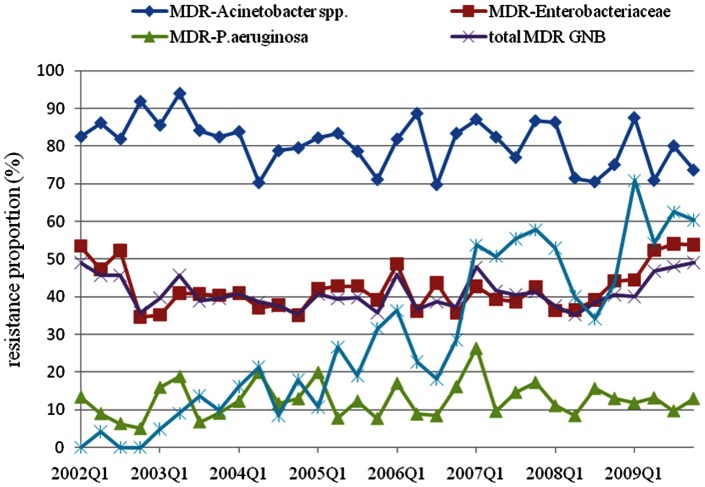
The resistance proportion of healthcare-associated infections caused by multidrug-resistant Gram-negative bacilli from 2002 to 2009. Abbreviations: CR =  carbapenems resistant; GNB =  Gram-negative bacilli; MDR =  multidrug-resistant; Q =  quarter.

During the 8-year study period, the incidences of GNB HAI and MDR-GNB HAI remained stable, while the incidence of overall HAI significantly decreased over time (Table 1). Despite the stable incidence of HAIs caused by MDR-GNB, MDR-Enterobacteriaceae, MDR-*P. aeruginosa* and MDR-*Acinetobacter* spp., HAIs due to CR-*Acinetobacter* spp. began to escalate in 2006 (*R = *0.84, *P* = 0.001) (Figure 1).

At the zero time lag, HAIs caused by CR-*Acinetobacter* spp. was found to positively correlate with the consumptions of carbapenems (*R = *0.79), extended-spectrum cephalosporins (*R = *0.86), fluoroquinolones (*R = *0.79), aminopenicillins/β-lactamase inhibitors (*R = *0.83), and piperacillin/tazobactam (*R = *0.63); negatively correlate with the consumptions of non-extended spectrum cephalosporins (*R = *−0.93), aminoglycosides (*R = *−0.93), narrow penicillins and aminopenicillins (*R = *−0.85), ureidopenicillins (*R = *−0.63), and folate pathway inhibitors (*R = *−0.86), and not correlate with consumption of glycopeptides (Table 3). The associations of the increase in HAI caused by CR-*Acinetobacter* spp. and consumptions of carbapenems, extended-spectrum cephalosporins, aminopenicillins/β-lactamase inhibitors, and fluoroquinolones could be traced to the time lag of −4 and were found in all positive time lags, while the association of the increase in HAI due to CR-*Acinetobacter* spp. and consumption of piperacillin/tazobactam could be found in the time lags of −3, −2, −1 and the zero time lag (Table 3).

**Table 3:** Correlations of antibiotic consumptions and healthcare-associated infections caused by carbapenem-resistant *Acinetobacter* spp. per 1,000 patient-days per quarter (2002 to 2009) and time lag effects of antibiotic consumptions.

Antibiotic	Time lag effect																		
	−4		−3		−2		−1		0		1		2		3			4	
	*R*	*P*	*R*	*P*	*R*	*P*	*R*	*P*	*R*	*P*	*R*	*P*	*R*	*P*	*R*	*P*	*R*		*P*
Non-extended-spectrum cephalosporins	−0.92	<0.01	−0.87	<0.01	−0.88	<0.01	−0.88	<0.01	−0.93	<0.01	−0.94	<0.01	−0.91	<0.01	−0.91	<0.01	−0.89		<0.01
Extended-spectrum cephalosporins	0.84	<0.01	0.78	<0.01	0.77	<0.01	0.83	<0.01	0.86	<0.01	0.82	<0.01	0.85	<0.01	0.83	<0.01	0.84		<0.01
Aminoglycosides	−0.91	<0.01	−0.91	<0.01	−0.93	<0.01	−0.92	<0.01	−0.93	<0.01	−0.92	<0.01	−0.91	<0.01	−0.91	<0.01	−0.91		<0.01
Natural penicillins and aminopenicillins	−0.67	<0.01	−0.77	<0.01	−0.78	<0.01	−0.79	<0.01	−0.85	<0.01	−0.81	<0.01	−0.81	<0.01	−0.78	<0.01	−0.76		<0.01
Ureidopenicillins	−0.56	<0.01	−0.51	<0.01	−0.53	<0.01	−0.50	<0.01	−0.63	<0.01	−0.62	<0.01	−0.74	<0.01	−0.79	<0.01	−0.79		<0.01
Carbapenems	0.72	<0.01	0.67	<0.01	0.74	<0.01	0.77	<0.01	0.79	<0.01	0.82	<0.01	0.76	<0.01	0.74	<0.01	0.71		<0.01
Fluoroquinolones	0.83	<0.01	0.87	<0.01	0.81	<0.01	0.82	<0.01	0.79	<0.01	0.74	<0.01	0.71	<0.01	0.68	<0.01	0.74		<0.01
Glycopeptides	0.09	0.65	−0.04	0.85	0.08	0.69	−0.02	0.89	0.13	0.49	0.09	0.60	0.04	0.84	−0.02	0.91	−0.05		0.79
Folate pathway inhibitors	−0.85	<0.01	−0.79	<0.01	−0.87	<0.01	−0.83	<0.01	−0.86	<0.01	−0.86	<0.01	−0.86	<0.01	−0.88	<0.01	−0.84		<0.01
Aminopenicillins/β-lactamase inhibitors	0.77	<0.01	0.75	<0.01	0.72	<0.01	0.76	<0.01	0.83	<0.01	0.78	<0.01	0.69	<0.01	0.76	<0.01	0.72		<0.01
Piperacillin/tazobactam	0.15	0.62	0.64	0.02	0.74	<0.01	0.59	0.02	0.63	<0.01	0.40	0.11	0.40	0.11	0.55	0.02	0.53		0.03
Overall antibiotics	−0.74	<0.01	−0.76	<0.01	−0.78	<0.01	−0.75	<0.01	−0.78	<0.01	−0.76	<0.01	−0.79	<0.01	−.083	<0.01	−0.82		<0.01

## Discussion

Our report explored the relationships among HAIs, MDR-GNB HAIs, and antibiotic consumptions, and provided insight into time lags regarding the emergence of HAIs due to CR-*Acinetobacter* spp. in association with the consumptions of a wide array of antibiotics. The rise in the incidence of HAIs depends mainly on infection control enforcement and antibiotic consumptions [21], [22]. Cross transmission between patients resulting from poorly persistent adherence to infection control practice causes bacterial dissemination, and high selective pressure resulting from the inappropriate and widespread use of antibiotics contributes to the development of MDR HAIs [21], [22].

During the 8-year study period, the trend in the overall HAI incidence was decreasing, while trends in GNB HAIs and MDR-GNB HAI in general remained stable, suggesting that the escalating incidence of HAIs due to CR-*Acinetobacter* spp. at KSCGMH was driven by antibiotic consumptions. Increasingly-used antibiotics at the study period included carbapenems, extended-spectrum cephalosporins, aminopenicillins/β-lactamase inhibitors, piperacillin/tazobactam, and fluoroquinolones. The increased consumptions of carbapenems, extended-spectrum cephalosporins, β-lactams/β-lactamase inhibitors, and fluoroquinolones have been reported to positively correlate with the development of MDR-GNB in a substantial number of studies [23], [24], [25], and the overconsumption of extended-spectrum cephalosporins (particularly ceftazidime) has been reported to potentially increase the prevalence of extended-spectrum β-lactamase (ESBL)-producing Enterobacteriaceae [25].

Piperacillin/tazobactam has been suggested, based on its potential ecological benefits, as an alternative to extended-spectrum cephalosporins for reducing the prevalence of ESBL-producing GNB in healthcare settings [24], [25], [26], [27]. Although various carbapenemase-associated genetic structures may be harbored in the isolates of *A. calcoaceticuse-A. baumannii* complex [28] and it was reported that prior exposure to piperacillin/tazobactam might render a patient subject to have higher chances of infections caused by CR-*Acinetobacter* spp. [5], [28], the association of increased use of piperacillin/tazobactam and the HAIs due to CR-*Acinetobacter* spp. was not found in this series (*P*>0.05 in all positive time lags) (Table 3).

In this series, non-extended cephalosporins, natural penicillins, aminopenicillins, ureidopenicillin and aminoglycosides were consumed less, and these antibiotics were negatively correlated with the increasing trend of HAIs caused by CR-*Acinetobacter* spp. The escalating trend of antibiotic resistance, pharmaceutical marketing practice, and changing physician perceptions may have contributed to the reduced consumptions of non-extended cephalosporins, and aminoglycosides. Potential side effects such as ototoxicity and renal toxicity may have been additional cause of the decline in the prescription of aminoglycosides. Because of the current high prevalence of antimicrobial-resistance [29], the usefulness of non-extended cephalosporins may be limited in the treatment of infections caused by GNB. However, our data suggests the need for strengthening the role of aminoglycosides in these circumstances. Aminoglycosides are reported to have a comparatively stable susceptibility rate among GNB that are acquired from either community or nosocomial sources [30], [31]. One study from a medical center disclosed that the empirical combined use of an anti-pseudomonal antibiotic (e.g., ceftazidime, cefpirome, cefepime, piperacillin/tazobactam, imipenem, or meropenem) and an aminoglycoside (i.e., gentamicin or amikacin) covered 85%−95% of the GNB isolated from the blood of neutropenic cancer patients [31], and another study suggested that patients with recent exposure to gentamicin or amikacin are at lower risk for acquiring bacteremia caused by *K. pneumoniae* isolates that simultaneously harbors plasmid-mediated ESBL and AmpC β-lactamase [32].

As HAIs caused by CR-*Acinetobacter* spp. escalates, clinicians must resort to broader-spectrum or newer antibiotics, furthering the vicious circle of increasing antibiotic resistance and demand for more effective antibiotic coverage for drug-resistant microbes, eventually resulting in the disastrous consequence of lacking effective antibiotics for MDR pathogens [1]. One limitation of this series is that DDD measurements did not assess antibiotic exposure at the level of individual patients where antibiotic selective pressure typically occurs [33], although the DDD measurements of antibiotic consumption are useful for comparison and benchmarking. In addition, this study was conducted at a single medical centre with its specific medical practice, infection control program and antibiotic prescribing patterns, and may therefore limits the generalizability when it comes to data interpretations.

In summary, our data demonstrated lacking significant association between the development of HAIs caused by CR-*Acinetobacter* spp. and the increased use of piperacillin-tazobactam, an antibiotic reported to have fewer negative impacts on microbiological ecology in hospitals [24], [25], [26], [27], . In addition, the results suggested antibiotic regimens with an aminoglycoside may help improve the incidence of HAIs due to CR-*Acinetobacter* spp. Given that the trend in overall HAIs decreased and trends in GNB HAIs and MDR-GNB HAIs remained stable, suggesting the effectiveness of infection control practice during the study period, the association of antibiotic consumptions and increasing HAIs caused by CR-*Acinetobacter* spp. underscores that the importance of antibiotic stewardship in the improvement of the trend of HAIs due to *Acinetobacter* spp. cannot be overemphasized.
